# Investigation of Radial Mixing Dynamics and Saturation Effect in Stirred Brown Rice Granular Systems Using Discrete Element Method

**DOI:** 10.3390/foods15020197

**Published:** 2026-01-06

**Authors:** Yawen Xiao, Yajuan Wang, Qianqian Yu, Minyue Sun, Xingeng Ni, Chunmeng Liu, Kexiang Zhang

**Affiliations:** School of Mechanical Engineering, Yangzhou University, Yangzhou 225127, China

**Keywords:** discrete element method, brown rice grains, stirring-shaft speed, radial mixing uniformity

## Abstract

In rice and food processing, spray mixing plays an essential role, with radial distribution uniformity serving as a key metric for assessing mixing performance. However, noticeable variations in the radial migration behavior of brown rice particles can occur even at identical stirring-shaft speeds, and the underlying mechanisms remain insufficiently understood. To clarify the influence of stirring-shaft speed on radial particle mixing, this study employed the discrete element method (DEM) to numerically simulate particle motion under different stirring-shaft speeds. The DEM model was experimentally validated by comparing simulated and measured particle volume proportions and power consumption, thereby ensuring the reliability of the numerical predictions. The results indicate a critical stirring-shaft speed of 20 rpm. Below this threshold, mixing uniformity increases with speed; above it, further increases yield negligible improvement. Analysis of different radial regions shows that selecting an appropriate stirring-shaft speed can effectively improve the mixing homogeneity across layers. In addition, the diffusion behavior of particles in different layers was analyzed, revealing that the diffusion capacity of each layer increases with stirring-shaft speed. These findings offer theoretical support for the optimal design and parameter setting of spray mixing equipment for brown rice and related food products.

## 1. Introduction

Rice (*Oryza sativa* L.) is a principal staple crop globally. A central aim of rice processing is to remove the bran layer from brown rice, as its presence detrimentally impacts cooking performance and sensory characteristics [1,2]. Rice milling is a traditional processing step that removes the bran layer from brown rice. However, during this process, the breakage rate and energy consumption are often excessively high, primarily due to the low moisture content of brown rice after storage. To address this, humidification and conditioning of the brown rice are required prior to milling [3].

**Figure 1:**
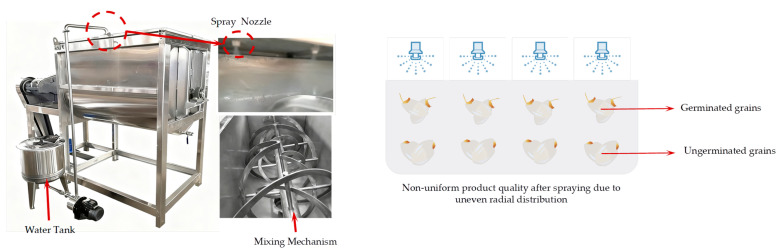
Non-uniform product quality after spraying due to uneven radial distribution (Left image courtesy of the internet).

The humidification device has a functional similarity to the agitation-mixing equipment employed in other industrial fields. Typically, humidification nozzles are installed above the brown rice mixing chamber [4]. Consequently, effective and uniform radial transport and mixing of brown rice grains, driven by the rotating shaft, are critical for achieving uniform humidification [5]. Apart from traditional rice milling, germinated brown rice has also garnered considerable research attention. During the germination process, the enzymes naturally released by the grain can not only break down or soften the bran layer but also enhance its nutritional value compared to white rice [6,7]. Similarly, the germination process of brown rice necessitates an agitation–mixing device fitted with spray nozzles above the mixing chamber. Inadequate radial movement and mixing of grains along the chamber’s vertical axis may result in some grains becoming over-saturated and developing mold, while others receive insufficient moisture, leading to reduced germination rates, as shown in Figure 1. Therefore, investigating the radial mixing mechanisms of brown rice particles within the mixing chamber is crucial for improving process uniformity and efficiency across operations such as humidification and germination. In this study, however, the analysis focuses specifically focuses on the dry granular mixing process.

Granular mixing technology is a key process in multiple industrial fields such as feed, chemical, food, and pharmaceuticals, and has garnered widespread research attention concerning mixing homogeneity. Domestic and international research teams have conducted extensive theoretical and experimental studies focused on the influence mechanism of stirring effects on the mixing homogeneity of granular materials [8,9]. For example, Lu et al. utilized a superquadric model to create non-spherical particles and examined the mixing performance and diffusion characteristics of cylindrical particles with varying aspect ratios in rotating drums at different stirring-shaft speeds [10]. Based on the discrete element method (DEM), Liu et al. established a radial mixing model for particles in a U-shaped container. They explored the radial mixing process and motion laws of ellipsoidal particles [11], uncovering how the mixing degree of these particles was affected by the number of rotations and the stirring-shaft speed. This study clarified the overall behavior of particle groups during mixing and pinpointed two distinct irregular motion patterns. Zhang et al. utilized the DEM soft-sphere model in the open-source software OpenFOAM to simulate the particle mixing behavior in a three-dimensional rotating drum at multiple stirring-shaft speeds. By incorporating a difference function, they quantitatively characterized the influence of stirring-shaft speed on mixing characteristics [12]. This study primarily analyzed the impact of stirring-shaft speed on the overall mixing characteristics of non-spherical particles, using the average positional difference calculated from radial and axial position differences to determine changes in the degree of mixing.

Previous studies have indicated that stirring-shaft speed could be one of the crucial factors influencing mixing. However, existing research has predominantly focused on the mechanisms influencing the macroscopic mixing characteristics of multi-component materials. Systematic investigations into how stirring-shaft speed affects radial mixing behavior across different vertical layers—particularly for mono-component, ellipsoidal grains such as brown rice—remain limited. Moreover, although stirring-shaft speed has been linked to overall mixing rates, whether a critical stirring-shaft speed exists beyond which improvements in radial mixing become negligible has not been clearly reported. These gaps limit the current understanding of layer-wise radial migration behavior in such systems.

To address this gap, the present study employs mono-component granular materials (brown rice) as the subject. A sphere-filling method is used to construct brown rice grain models with identical morphology, and DEM is applied to simulate the mixing characteristics of brown rice grains in a U-shaped container at different stirring-shaft speeds. This study explores the influence mechanism of stirring-shaft speed on the radial mixing uniformity of mono-component granular materials. It aims to offer insights into mixing behavior and facilitate the selection of suitable operating parameters in brown rice handling processes, thereby providing valuable references for optimizing grain spray-mixing equipment and associated processes.

## 2. Numerical Simulation Methods

**Figure 2:**
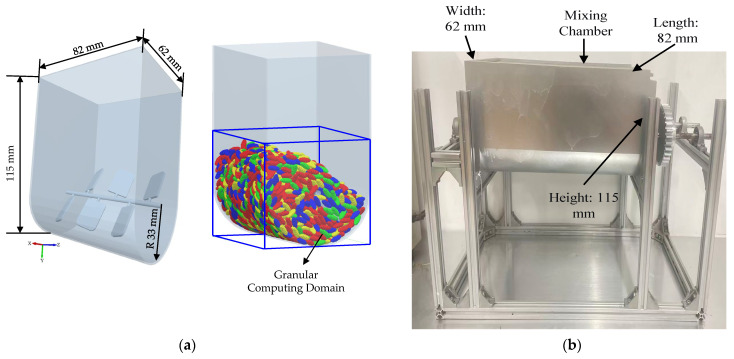
(**a**). Geometry of the U-shaped container used in DEM simulations; (**b**). Structural support frame of the laboratory-scale U-shaped mixing chamber used for experimental validation.

**Figure 3:**
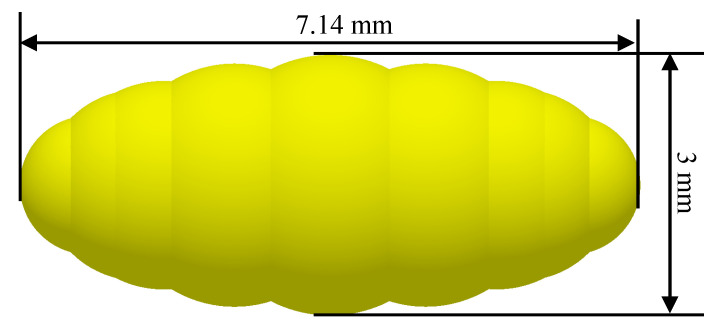
Brown rice model.

This study utilized numerical simulation methods to explore the motion characteristics of uniformly sized brown rice within a U-shaped container, with particular emphasis on analyzing how different stirring-shaft speeds influence the radial mixing degree of ellipsoidal brown rice particles. The U-shaped container consists of a U-shaped outer shell and internal stirring blades, as shown in Figure 2a. The outer shell length, width, and inner diameter of the bottom of the U-shaped container are 115 mm, 82 mm, and 33 mm, respectively. The stirring shaft within the U-shaped container is symmetrically equipped with six blades arranged in an alternating upper-lower pattern. The blades are inclined at a 30° angle relative to the horizontal plane, measuring 20 mm in length, 14 mm in width, and 1.5 mm in thickness. The distance between adjacent stirring blades is 12.9 mm. The simulated particles were modeled after brown rice grains after hulling, which approximate an ellipsoidal shape in appearance. Through repeated measurements, the mean lengths of their major and minor semi-axes were determined to be 7.14 mm and 3 mm, respectively. Based on these parameters, the ellipsoidal particles were modeled using the ‘sphere filling method’, as illustrated in Figure 3.

**Figure 4:**
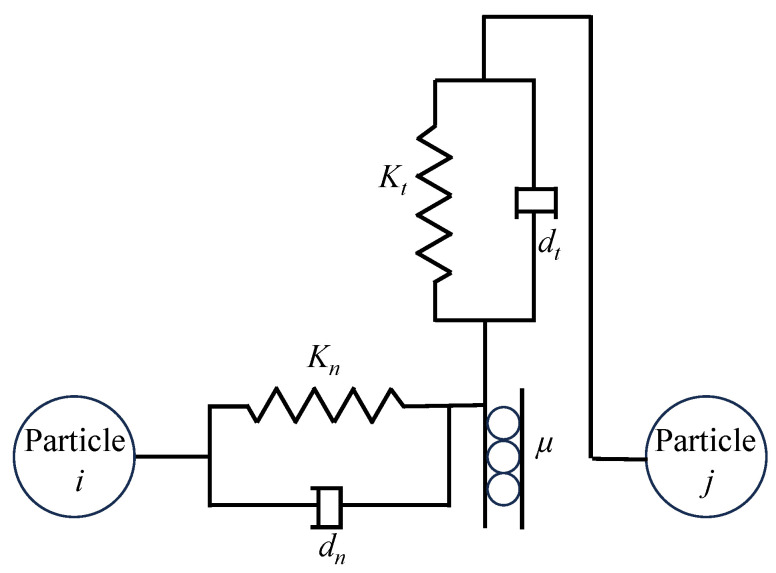
Contact mechanics model of Hertz–Mindlin.

This study models ellipsoidal brown rice particles as dry particles, in which the effects of liquid bridge force and adhesive force are negligible because there is no liquid around the particle contact points. Therefore, this paper employs the Hertz–Mindlin contact model and adopts the soft-sphere approach. In this model, the forces between ellipsoidal brown rice particles are decomposed into normal and tangential components: the normal force includes elastic and damping forces, while the tangential force includes elastic force, damping force, and Coulomb friction force. Figure 4 shows a schematic diagram of the contact mechanics model for ellipsoidal particles.

Based on the aforementioned contact mechanics model, when conducting force analysis on the brown rice particle model within the U-shaped container, the forces acting on particle *i* consist of three components: its own gravitational force under the gravity field, the normal contact force from collisions with other particles, and the tangential contact force from such collisions. The motion of the particles follows Newton’s second law, and the equations of motion are presented as follows:

(1) $$m_{i}\frac{dv_{i}}{dt}=m_{i}g+\sum_{j=1}^{n_{i}}\left(F_{n}+F_{n}^{d}+F_{t}+F_{t}^{d}\right),$$

In this Equation (1), *v* denotes the velocity of the brown rice particle (m/s) and *m* denotes its mass (kg), and *n_i_* represents the total number of brown rice particles in contact with particle *i*. The terms $F_{n}$, $F_{n}^{d}$, $F_{t}$ and $F_{t}^{d}$ refer to the normal contact force (N), normal damping force (N), tangential contact force (N), and tangential damping force (N) between brown rice particles, respectively. To accurately evaluate energy transfer and dissipation characteristics during particle collisions, the model decomposes the interparticle forces into mutually perpendicular normal and tangential components based on the principle of vector decomposition.

The normal contact force is given by Equation (2):

(2) $$F_{n}=\frac{4}{3}E*\sqrt{R*\alpha^{3}},$$

where *E* represents the elastic modulus of the ellipsoidal brown rice particles (MPa); *R* denotes the equivalent curvature radius at the contact point between two ellipsoidal brown rice particles, which can be calculated from their respective curvature radii (mm); and *α* represents the radius of the circular contact area formed during contact between the ellipsoidal particles (mm).

The normal damping force is given by Equation (3):

(3) $$F_{n}^{d}=-2\sqrt{\frac{5}{6}}\frac{\ln\varepsilon }{\sqrt{\ln^{2}\varepsilon +\pi^{2}}}\sqrt{S_{n}m*v_{n}^{\mathrm{rel}}},$$

where ε represents the coefficient of restitution, *S_n_* denotes the normal stiffness (N/mm), which characterizes the ability of an ellipsoidal particle to resist deformation in the normal direction and is related to the elastic modulus and particle geometry *m* is the equivalent mass of the two colliding ellipsoidal particles (kg), and $v_{n}^{\mathrm{rel}}$ is the relative velocity of the contacting particles in the normal direction (m/s).

The tangential contact force $F_{t}$ and tangential damping force $F_{t}^{d}$ acting on the ellipsoidal particles in the model are given by Equation (4) and Equation (5), respectively:

(4) $$F_{t}=-S_{t}\delta ,$$

(5) $$F_{t}^{d}=-2\sqrt{\frac{5}{6}}\frac{\ln\varepsilon }{\sqrt{\ln^{2}\varepsilon +\pi^{2}}}\sqrt{S_{t}m*v_{t}^{rel}},$$

where *S_t_* denotes the tangential stiffness coefficient (N/mm, calculated from particle radius, elastic modulus, and collision overlap), δ represents the tangential overlap (mm), and $v_{t}^{\mathrm{rel}}$ indicates the relative velocity of the contacting particles along the tangential direction (m/s). Compared to the normal force, the tangential force applied to ellipsoidal brown rice particles additionally induces a rolling friction torque and a tangential torque. The rotational equation is given as follows:

(6) $$I_{i}\frac{dw_{i}}{dt}=\sum_{j=1}^{n_{i}}\left(T_{t}+T_{r}\right),$$

where *I_i_* is the moment of inertia of the particle (kg·mm^2^), *T_t_* is the tangential moment (N·mm), and *T_r_* is the rolling friction moment (N·mm). The physical parameters related to particle collision and motion in the simulation system are listed in Table 1, and the simulation parameters were selected with reference to Ref. [13].

The Hertz–Mindlin (no-slip) contact model was adopted for its theoretical rigor and widespread validation in simulating dry, cohesionless granular flows. This model is grounded in classical contact mechanics and is particularly suitable for systems where plastic deformation and adhesion are negligible—conditions that align with the behavior of stiff, low-moisture agricultural grains like brown rice. By resolving collisions through elastic and damping forces in both normal and tangential directions, the model captures key energy dissipation mechanisms, such as inelastic collisions and frictional sliding, which govern the collective flow and mixing behavior of granular assemblies. The selection of model parameters (Table 1) was guided by experimentally measured properties of brown rice, ensuring that the simulated contact dynamics realistically represent the physical interactions between grains, and between grains and equipment surfaces. This physics-based approach enhances the credibility of the DEM predictions when extrapolating beyond the experimentally validated conditions.

Simulations were conducted using the commercial DEM software EDEM 2.7^®^. An explicit integration time step of 6.31066 × 10^−6^ s was used to ensure numerical stability. A grid sensitivity analysis confirmed that the results were independent of the background computational grid size when set to 3 times the smallest particle radius. The simulations, involving approximately 3364 particles, were performed on a workstation with an Intel^®^ Core™ i9-13900K CPU and 64 GB RAM, with each 30-s physical process requiring approximately 12 h of computational time. In the DEM simulation, a particle factory is established beneath the opening of the U-shaped container. Subsequently, ellipsoidal brown rice particles descend under the influence of gravity and accumulate. Once the material bed height reaches 43 mm, the particle factory is removed and the system remains stationary for 0.5 s to allow the particle layer to stabilize. The stirring shaft then begins to rotate. By varying the stirring-shaft speed (set at 10, 20, 30, 40, and 50 rpm), the influence of stirring-shaft speed on the mixing characteristics was investigated.

## 3. Results and Discussion

### 3.1. Experimental Validation of the Discrete Element Model

To assess the accuracy of the numerical simulations, a dedicated laboratory-scale experimental setup was constructed for model validation (Figure 2b). The setup consisted of a geometrically identical U-shaped mixing chamber, fabricated from stainless steel, mounted on a rigid support frame. A variable-speed electric motor equipped with a digital controller and a built-in torque sensor was connected to the stirring shaft. This configuration allowed for precise control of the stirring-shaft speed and direct measurement of the shaft torque during operation.

**Figure 5:**
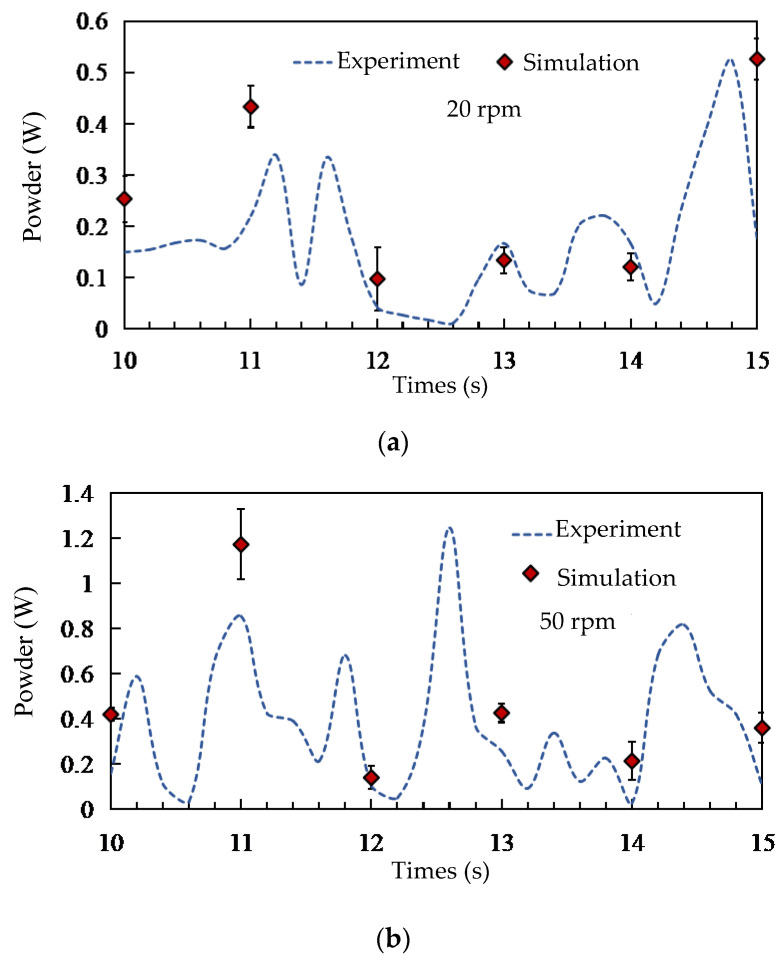
Comparison of simulated and experimentally measured power consumption of the stirring shaft at two stirring-shaft speeds: (**a**) 20 rpm and (**b**) 50 rpm.

The validation procedure comprised two parallel approaches: power consumption comparison and particle distribution analysis. For the power consumption validation (as shown in Figure 5), brown rice grains were loaded into the chamber with an initial filling ratio of 15%. The stirring shaft was operated at two representative speeds, 20 rpm (low-speed regime) and 50 rpm (high-speed regime). Each speed condition was repeated for five independent experimental runs to account for stochastic variability. In each run, after the motor reached steady-state operation, the instantaneous torque and rotational speed were recorded at a sampling frequency of 10 Hz over a duration of 15 s. The instantaneous power was calculated. The no-load power (measured by operating the empty mixer) was subtracted from the total power to obtain the net power consumed solely for particle mixing. The final experimental power value for each speed was reported as the average net power over the stable measurement period across all five replicates. In the corresponding DEM simulations, the power was calculated using the power calculation method proposed by Jayasundara et al. [14,15], as expressed by the following formula:

(7) $$E=\int_{0}^{t}M_{r}\omega dt$$

Here, *E* denotes the energy consumption, *M_r_* denotes the instantaneous torque of the screw shaft at each time step, and *t* denotes the total duration of the mixing process.

**Figure 6:**
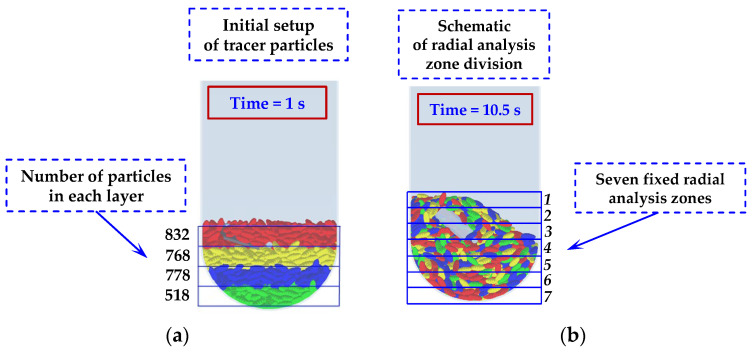
Schematic of the methodology for tracking particle mixing: (**a**) Initial coloring of tracer particles into four radial layers; (**b**) Division of the particle bed into seven fixed radial analysis zones for quantitative evaluation.

For the particle distribution validation, a tracer-based sampling method was employed. In the experiment, four distinct batches of rice were physically dyed with different, non-transferable food-safe colors (red, yellow, blue, green) to serve as tracers, corresponding to the four colored particle groups defined in the simulation (Figure 6a). These batches were initially layered radially within the chamber before mixing. After operating the mixer at a specified speed (20 or 50 rpm) for a pre-determined duration to reach a statistically steady mixing state, the process was halted. A multi-compartment sampling probe was then inserted into specific, pre-defined locations within the chamber to extract simultaneous samples from different radial zones. The collected grains from each compartment were manually separated by color, weighed, and their mass fractions were calculated. This sampling and weighing procedure was repeated three times for each operational condition to obtain an average mass proportion distribution. In the DEM simulation, virtual particle samples were extracted from the exact same volumetric regions as the physical sampling locations. The number of particles belonging to each tracer group within these virtual samples was counted and converted into a volume proportion for direct comparison with the experimental mass proportions, assuming uniform particle density.

**Figure 7:**
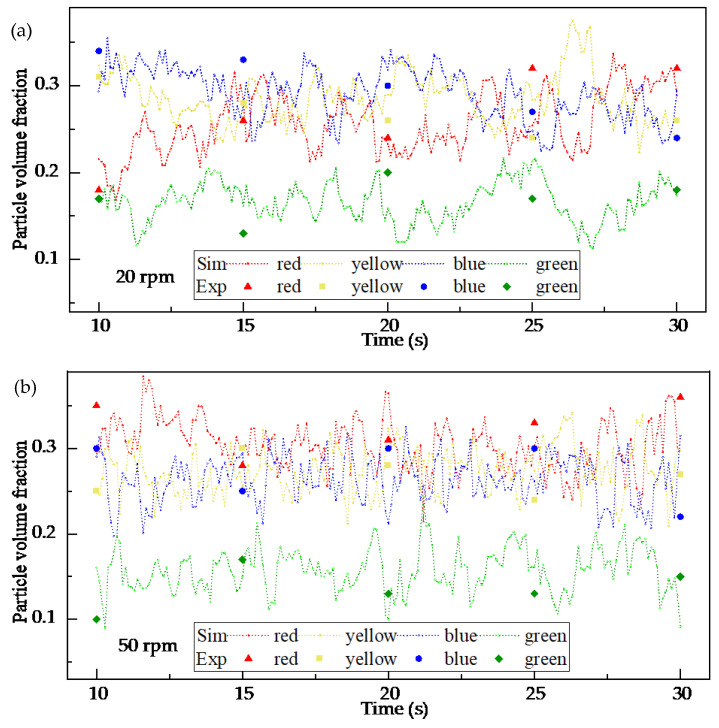
Experimental validation of the DEM model based on particle volume proportion at two stirring-shaft speeds: (**a**) 20 rpm and (**b**) 50 rpm.

The results of these validation efforts are presented in Figure 5 and Figure 7. Figure 5 shows a close agreement between the simulated and experimentally measured power consumption trends and magnitudes at both speeds. Minor deviations fall within the range expected from the inherent randomness of granular flows and measurement uncertainties. Figure 7 demonstrates that the simulated radial distribution of tracer groups matches the experimental findings, capturing the same distribution trends and regional variations. The consistency achieved through these two independent validation metrics confirms that the DEM model, with its calibrated parameters, reliably replicates the key mechanical and kinematic aspects of the real mixing system. This validated model forms the basis for the subsequent numerical investigation of parameters and mechanisms.

### 3.2. Analysis of Particle Radial Mixing Motion During the Stirring Process

**Figure 8:**
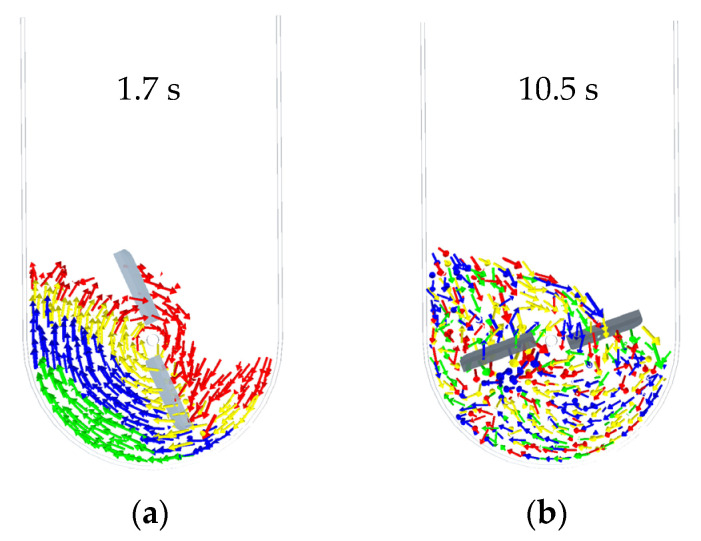
(**a**) Morphology of particle assembly at 1.7 s, (**b**) morphology of Particle assembly at 10.5 s.

In order to elucidate the mechanism of radial particle mixing, the motion of tracer particles was analyzed. The simulation results indicate that the particles exhibit large-scale motion as the mixing shaft rotates. Through contact, mixing, and collision, the four groups of tracer particles achieve a uniform distribution and establish a stable flow (Figure 8a,b).

**Figure 9:**
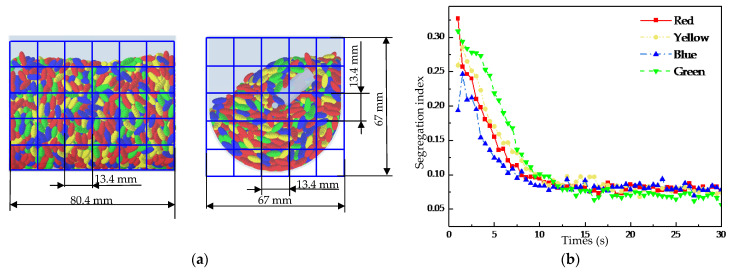
(**a**) The grids on the XZ-plane (**left**) and grids on the YZ-plane (**right**), (**b**) Variation of the segregation index with time at 20 rpm.

A prerequisite for analyzing the radial mixing process is to determine whether the particles have achieved complete mixing. To this end, the segregation index was utilized, following the method described in the literature [13], to evaluate the overall mixing process of the tracer particles within the U-shaped container. For the calculation of the segregation index, the cross-sections of the U-shaped container were discretized into grids: a 5 × 8 grid on the YZ plane and a 6 × 8 grid on the XZ plane (Figure 9a), with each grid cell (13.4 mm on each side) constituting a sample. Due to the geometry of the container, the external location of some sampling regions, and the absence of particles in the upper part, there were significant variations in the number of particles per sample. To address this, a weighting method was adopted. This approach ensured that samples with a larger proportion of particles were assigned a greater weight. The formula for the segregation index S is as follows:

(8) $$S=\sqrt{\frac{1}{k}\sum_{i=1}^{N_{s}}k_{i}{\left(a_{i}-\bar{a}\right)}^{2}},$$

(9) $$k=\sum_{i=1}^{N_{s}}k_{i},$$

(10) $$k_{i}=N_{i}/N_{t},$$

where: *N_s_* is the total number of samples; *a_i_* is the volume fraction of tracer particles within sample *i*. $\bar{a}$ is the volume fraction of the tracer particle group relative to the total particles in the U-shaped container; *N_i_* is the total number of particles within sample *I*; *N_t_* is the total number of particles across all samples; *k_i_* is the weighting factor for an individual sample *i*.

Figure 9b presents the curve of the segregation index versus time at 20 rpm, with a smaller value indicating a more homogeneous mixture of the tracer particle groups. The plot shows that during the initial phase (1–7.5 s), the segregation index dropped rapidly in response to the rotation of the stirring shaft. This behavior is attributed to the rapid motion of the tracer particles driven by the stirring shaft in the initial stage, which facilitated mutual penetration and contact among different groups, thereby achieving highly efficient mixing. In the subsequent phase (7.5–15 s), the mixing efficiency declined with ongoing homogenization, and the segregation index’s downward trend moderated and leveled off. Finally, over 15–30 s, the index remained invariant with the rotation of the stirring shaft, suggesting that a state of uniform mixing had been attained for the tracer particles in the U-shaped container.

Based on the foregoing analysis, this study further evaluated the mixing homogeneity by acquiring particle motion parameters from 15 to 30 s—with 15 s as the start time and a 0.5-s sampling interval—to investigate the radial mixing behavior of ellipsoidal particles during stirring. In this study, the proportion of each tracer particle group within each radial analysis area was calculated. We used the degree of its consistency with the group’s initial proportion as the evaluation metric. When the stirring shaft rotates within the U-shaped container, it drives the particle assembly. However, due to inertial effects, particles near the upper part section of the mixing chamber tend to climb unilaterally along the wall surface. As the stirring shaft keeps rotating, these particles periodically collapse, resulting in an uneven distribution of tracer particles across different analysis layers and showing dynamic fluctuations. Moreover, due to the structural differences of the U-shaped container, each group of tracer particles also shows a non-uniform distribution in the initial state. These three phenomena can all influence the assessment of mixing homogeneity. Therefore, to reduce the impact of these factors, this study introduces a quantitative indicator r/R. Here, r represents the proportion of a specific group of tracer particles relative to the total number of particles in a given radial analysis region, and R represents the proportion of each group of tracer particles relative to the total number of particles in the U-shaped container under initial conditions. This indicator can be used to evaluate the distribution uniformity of particle mixing in different radial regions, and its calculation is given by Equations (11) and (12):

(11) $$r=\frac{n_{x}^{i}}{n_{x}},$$

The proportion *r* of a specific tracer particles group is defined as the ratio of the number of tracer particles from group *i* (*i* = 1, 2, 3, 4) present in a given particle layer to the total number of particles within the rectangular analysis zone of that layer.

(12) $$R=\frac{N_{i}}{N_{0}},$$

The proportion *R* of a given group of tracer particles is defined as the ratio of the number of particles from group *i* (*i* = 1, 2, 3, 4) in each radial analysis region at the initial moment to the total number of particles in all analysis regions. Based on this indicator, statistical analysis was conducted on the distribution of tracer particles across various analytical regions to gain deeper insights into the movement patterns of particles in the radial direction and their dispersion uniformity during the mixing process.

**Figure 10:**
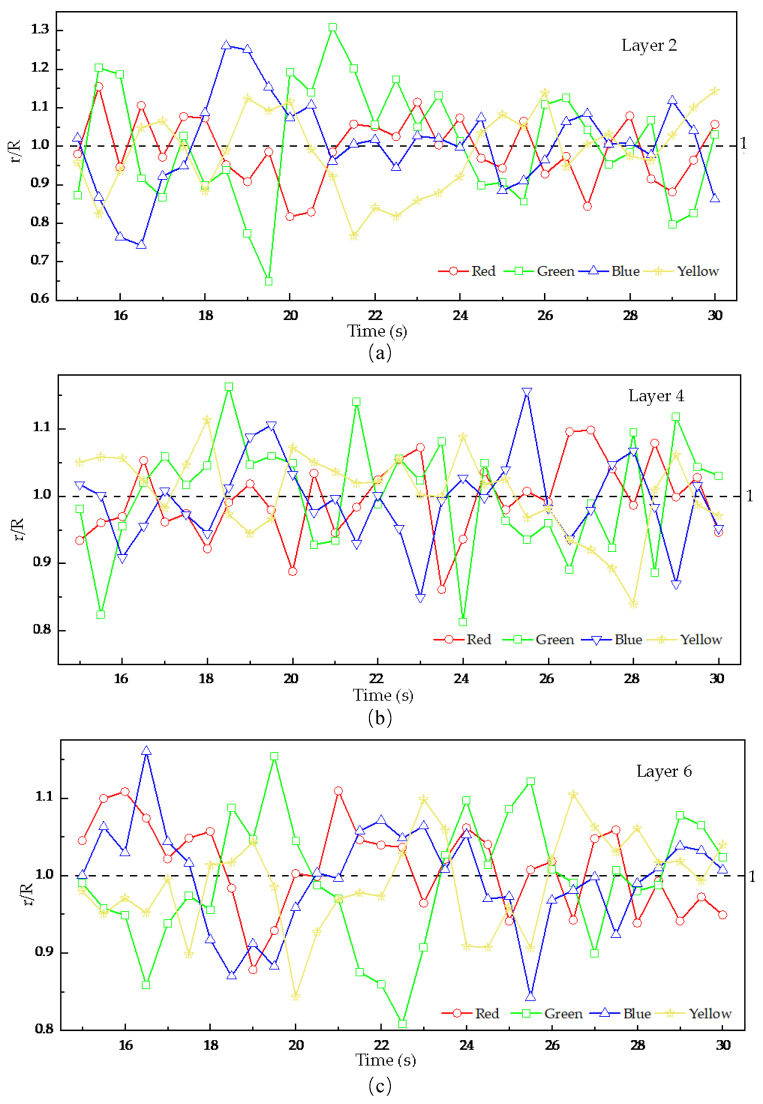
Radial mixing uniformity of particles in different granular layers as a function of time, (**a**) the second layer, (**b**) the fourth layer, (**c**) the sixth layer.

**Figure 11:**
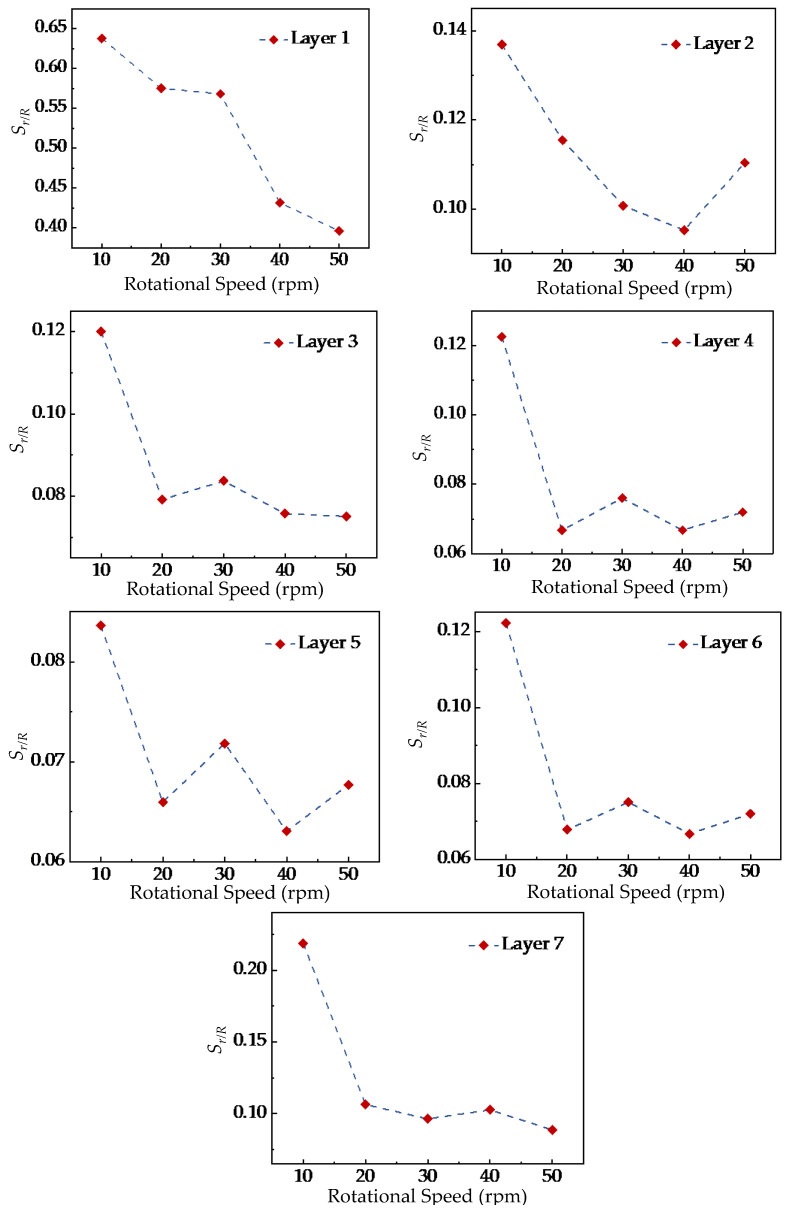
Variation of mixing uniformity characteristics with stirring-shaft speed in different radial analysis regions.

As shown in Figure 10, focusing on the second, fourth, and sixth analysis layers, the figure presents the temporal evolution of radial distribution uniformity for the four groups of tracer particles at a stirring-shaft speed of 20 rpm, using the previously defined *r/R* indicator, with a theoretical baseline value of 1, which indicates that the distribution of tracer particles becomes more uniform across analysis regions as the *r/R* value approaches this baseline. As can be seen from Figure 11, the *r/R* values for all tracer groups fluctuate around 1 with gradually decreasing amplitude, indicating that the radial uniformity progressively improves as the stirring-shaft speed increases. A comparative analysis of the mixing degree of tracer particles across different layers in Figure 11 reveals that the *r/R* ratio of the fourth-layer tracer particles exhibits smoother fluctuations than those in the second and sixth layers. This indicates that the radial distribution uniformity within the fourth analysis region is significantly better. Combined with the division of analysis zones in this study (as shown in Figure 8b), the fourth layer corresponds to the root of the stirring blades, whereas the second and sixth layers cover the upper–middle blade sections. This suggests that the location of the blade relative to the particles influences the radial mixing behavior, which holds implications for stirring-blade design. Moreover, the second layer lies within the influence zone of the cascading flow near the upper free surface. As shown in Figure 8b, the periodic collapse of this cascading flow, formed by the rotation of the stirring shaft, is likely the reason for the excessive fluctuations in the quantitative indicator *r/R* values of the tracer particles in the second layer [13].

### 3.3. Effect of Stirring-Shaft Speed on Radial Mixing Uniformity of Particles

Previous studies have indicated that the overall mixing uniformity of different particle groups is influenced by factors such as stirring-shaft speed, number of stirring blades, and particle properties [16,17,18]. For the ellipsoidal particles of identical composition located in different layers in this study, the relationship between their radial mixing homogeneity and the aforementioned influencing factors has not been clearly established. The analysis above revealed that the differences in radial mixing behavior fluctuations primarily originate from the driving capacity of the stirring blades and the periodic collapse of the upper “cascading flow,” while both potential causes are closely related to the stirring-shaft speed of the stirring shaft. Accordingly, in this study, while maintaining other operational parameters unchanged, numerical simulations were conducted under five stirring-shaft speeds (10 rpm, 20 rpm, 30 rpm, 40 rpm, and 50 rpm), and the variations in the quantitative indicator *r/R* were analyzed.

As shown in Figure 11, the quantitative indicator r/R of tracer particles in each layer fluctuates around a stable value of 1, where the fluctuation amplitude reflects the degree of radial mixing uniformity. To avoid repeatedly describing similar fluctuation patterns across layers, the overall trend is summarized here: smaller amplitudes indicate improved uniformity, while larger amplitudes reflect poorer mixing. To further quantify the differences in particle mixing uniformity under different stirring-shaft speeds, this study selected the time period from 15 s to 30 s, extracted the data for each speed, and calculated the standard deviation *S_r/R_* to reflect the influence of stirring-shaft speed on radial mixing effectiveness.

**Figure 12:**
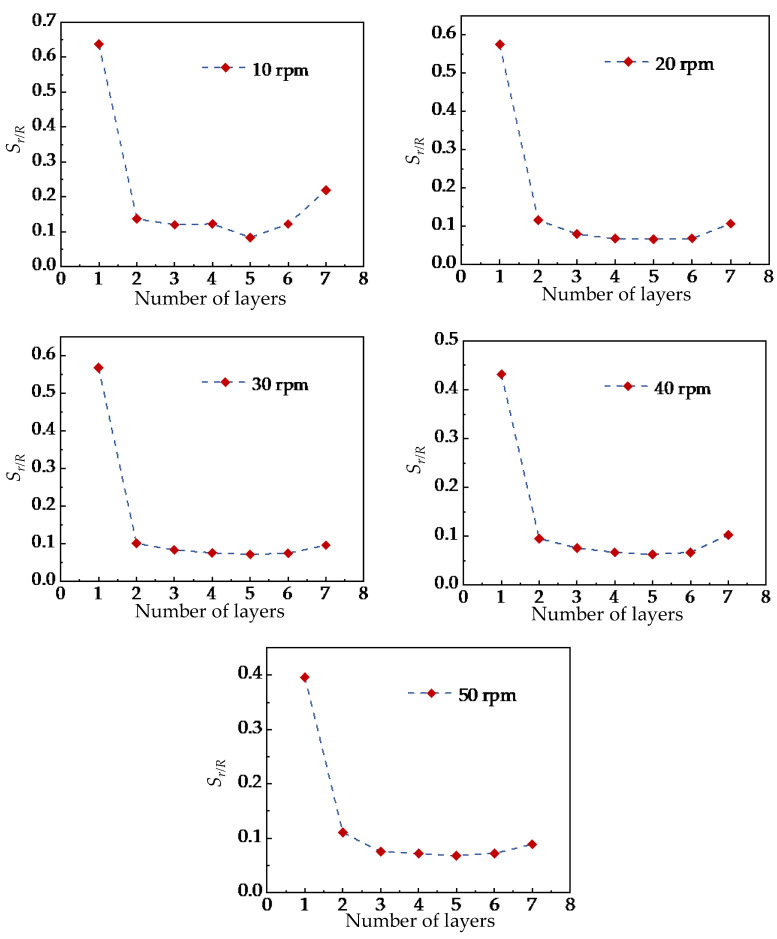
Differences in mixing uniformity of particles in different radial analysis regions at the same stirring-shaft speed.

Figure 12 shows the variation of *S_r/R_* with the stirring-shaft speed of the stirring shaft in different radial analysis regions. The results indicate that the influence trends of different stirring-shaft speeds are generally consistent across all analysis regions. The value of *S_r/R_* decreases initially and then stabilizes as the stirring-shaft speed increases. When the stirring-shaft speed reaches 20 rpm, further increases in speed have no significant effect on the particle mixing homogeneity. These findings are consistent with Liu et al. [13], who reported that stirring-shaft speed mainly affects the mixing rate rather than the final uniformity. Based on this agreement, the present study identifies 20 rpm as a critical stirring-shaft speed beyond which the improvement in mixing uniformity becomes negligible. By comparing the differences in radial mixing homogeneity before and after reaching the critical stirring-shaft speed across various analysis layers, it was found that the differences in Layers 1 and 2 were significantly greater than those in Layers 3 to 6. This phenomenon is likely attributed to the more pronounced influence of the ‘cascading flow’ on Layers 1 and 2. Nevertheless, the findings also indicate that this cascading-flow effect does not amplify with speed increase, offering valuable insights for preventing superfluous power consumption in practical mixer design.

Meanwhile, this study also observed that the standard deviation value Sr/R of Layer 7 was significantly higher than that of Layers 3 to 6, suggesting that the mixing uniformity of tracer particles in Layer 7 was lower than in the other layers. Furthermore, the difference in Layer 7 gradually increased as the stirring-shaft speed rose from 20 to 50 rpm and then slowly decreased. This result indicated that although the stirring-shaft speed had a relatively minor effect on the overall radial mixing uniformity of particles, it still influenced the mixing degree of tracer particles in Layer 7. Combined with the location of the analyzed region, Layer 7 is situated at the bottom of the mixing tank, adjacent to the top of the stirring blades, which results in a weaker upward transport capacity of particles. Therefore, improving the upward transport in this region is essential, and appropriately increasing the length of the stirring shaft or adjusting the size of the top impeller blades may enhance the mixing effectiveness in Layer 7, providing useful guidance for practical mixer design.

To more intuitively demonstrate the differences in particle mixing uniformity across different radial analysis zones, Figure 12 further illustrates the variation trend of *S_r/R_* within each radial analysis zone at the same stirring-shaft speed. The results reveal that at the same stirring-shaft speed, the differences in particle mixing uniformity across various radial analysis zones remain consistent, with the radial mixing uniformity at the top and bottom layers being significantly lower than that of the intermediate layers. These consistent trends suggest that the flow structures near the free surface and the tank bottom inherently limit mixing performance in these regions. Therefore, during the design of the mixing chamber, the formation of a ‘cascading flow’ at the top layer should be avoided, and the upward transport capacity of particles from the bottom to upper layers should be enhanced to improve overall mixing efficiency in practical applications.

### 3.4. Effect of Stirring-Shaft Speed on Radial Dispersion of Particles

According to previous findings, when the stirring-shaft speed of the mixing shaft reached 20 rpm, the radial mixing uniformity of the tracer particles had already stabilized. Further increasing the stirring-shaft speed showed no significant improvement in the radial mixing effect of particles within the analysis zone. To elucidate the fundamental mechanism behind the saturation phenomenon of the ‘stirring-shaft-speed–mixing-effect’, this study introduces the dispersion coefficient *D* as a quantitative evaluation metric to thoroughly investigate the influence of stirring-shaft speed on the radial mixing performance of tracer particles across different layers, with its calculation formula given as Equation (13).

(13) $$D=\frac{1}{N}\sum_{i=1}^{n}\frac{{\left(\Delta r_{i}\right)}^{2}}{2\Delta t},$$

where: *D* represents the mean diffusion coefficient of all particles (mm^2^/s); *N* denotes the total number of particles (dimensionless); Δt indicates the time interval for particle motion (s); and $\Delta r_{i}$ represents the displacement magnitude of particle *i* over the time interval Δt (mm).

**Figure 13:**
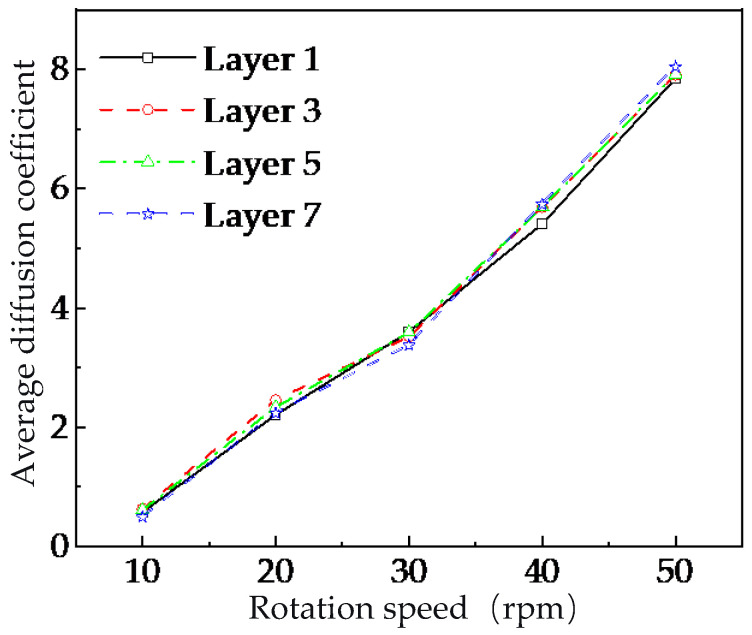
Mean values of dispersion coefficients in the radial analysis region of each layer at different stirring-shaft speeds.

To facilitate comparison of the mean diffusion coefficients of tracer particles across different layers in the U-shaped container under various stirring-shaft speeds, this study selected tracer particles during the stable flow period (15–30 s) and calculated the average diffusion coefficient within radial analysis zones. As shown in Figure 13, taking Layers 1, 3, 5, and 7 as examples, the diffusion capacity of tracer particles with-in each radial analysis zone increases with the stirring-shaft speeds in the U-shaped container. However, this phenomenon contradicts previous findings which demonstrated that the radial mixing performance does not improve further when the stirring-shaft speed exceeds 20 rpm.

**Figure 14:**
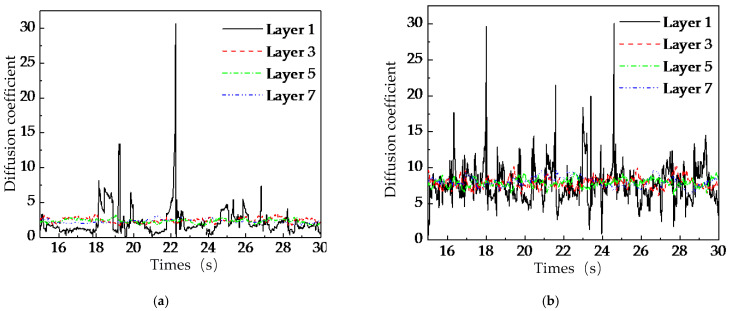
Temporal variation of particle diffusion coefficients in different radial analysis regions: (**a**) 20 rpm, (**b**) 50 rpm.

To further investigate the mechanism behind the ‘stirring-shaft-speed–mixing-effect’ saturation phenomenon, this study conducted an in-depth analysis of the temporal evolution of particle diffusion coefficients within radial regions of the U-shaped container under two operational conditions of stirring-shaft speed: 20 rpm and 50 rpm, as shown in Figure 14. Experimental results demonstrate that although the diffusion coefficient of particles in each radial analysis zone shows a significant increasing trend with the rise of the stirring shaft speed, its fluctuation amplitude also increases accordingly. Combined with the observations from Figure 12, it can be seen that due to the influence of the ‘cascading flow’, the mixing uniformity of particles in Layer 1 is lower than that in the other layers. The quantitative analysis in Figure 14a further reveals that the instability of the diffusion coefficient caused by this effect may be a key factor weakening radial mixing effectiveness. As shown in Figure 14b, when the stirring-shaft speed of the stirring shaft reaches 50 rpm, the particle diffusion coefficient increases, indicating enhanced radial diffusion capacity. However, the diffusion coefficient also exhibits greater instability, and the radial mixing uniformity of particles does not improve significantly. It can be inferred that the intensified fluctuations in the diffusion coefficient may have an inhibitory effect on the particle mixing process.

**Figure 15:**
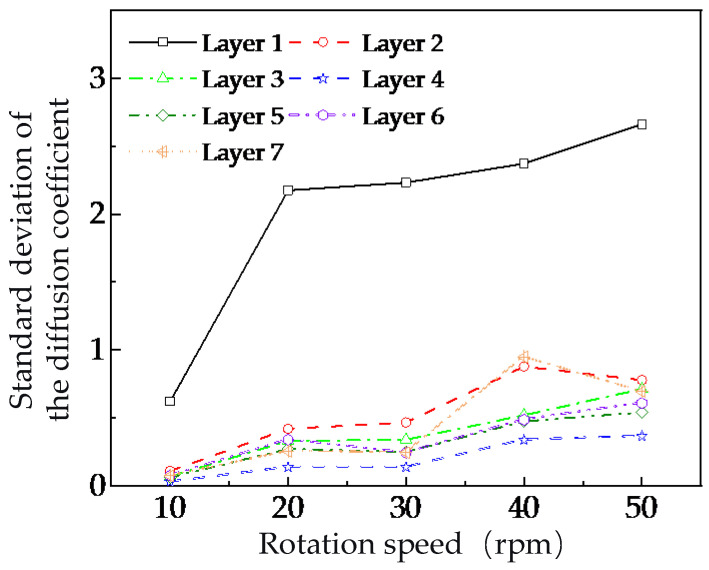
Variation of particle diffusion coefficients in different radial analysis regions.

This study selected diffusion coefficient data of tracer particles from each analysis layer within the time interval from 15 s to 30 s and calculated their standard deviations to quantitatively evaluate the fluctuations of particle diffusion coefficients in different analysis zones under various stirring-shaft speeds. As shown in Figure 15, with the increase of the stirring shaft’s stirring-shaft speed, the fluctuation level of particle diffusion coefficients in all layer increases, with more significant increases observed in Layers 1 and 7. This suggests that higher stirring-shaft speeds intensify the fluctuations of particle diffusion coefficients in both the top and bottom layers, thereby reducing mixing effectiveness.

## 4. Discussion

This study employed the discrete element method (DEM) to systematically investigate the radial mixing behavior of dry brown rice particles in a U-shaped mixer. The primary objective was to elucidate the influence of stirring-shaft speed on mixing uniformity and to identify any saturation effects. The findings reveal a critical stirring-shaft speed of approximately 20 rpm. Below this threshold, increasing speed significantly enhances radial mixing uniformity. However, beyond 20 rpm, further increases yield diminishing returns, indicating a saturation point in mixing effectiveness.

This saturation phenomenon can be attributed to two competing mechanisms. On the one hand, higher stirring-shaft speeds promote stronger particle diffusion, as evidenced by the increasing mean diffusion coefficients (Figure 13). On the other hand, these higher speeds simultaneously amplify transient fluctuations in particle motion, particularly within the upper cascading-flow zone and the bottom layer near the blade roots (Figure 14 and Figure 15). The intensified instability of particle trajectories at elevated speeds counteracts the potential benefits of enhanced diffusion, leading to the observed plateau in overall mixing homogeneity. This behavior aligns with previous studies suggesting that mixing performance in similar systems is governed more by the total number of rotations than by speed alone. Our results extend this understanding by revealing the underlying radial-layer mechanisms responsible for the saturation effect. It is crucial to reiterate that the present DEM model and analysis focus exclusively on dry granular mixing. The absence of liquid bridges and cohesive forces allows for the isolation and fundamental study of particle transport mechanics driven solely by mechanical agitation. This foundational knowledge is directly applicable to processes where dry mixing is a distinct phase. Nevertheless, the insights gained have significant implications for more complex industrial processes, such as spray-based conditioning or germination of brown rice, where uniform radial distribution of grains is a critical prerequisite for subsequent uniform liquid application. The identification of a critical speed and the understanding of persistent mixing weaknesses in the top and bottom layers provide a theoretical framework for optimizing operational parameters in such systems. For instance, operating near the identified critical speed (~20 rpm) may offer an optimal balance between achieving satisfactory mixing uniformity and minimizing unnecessary energy input. Furthermore, the results underscore that geometric constraints, rather than kinetic energy input alone, limit mixing performance in certain regions. The persistent non-uniformity in the top layer (driven by the unstable cascading flow) and the bottom layer (due to weak upward transport) suggests that structural modifications, rather than further increases in stirring-shaft speed, are necessary for substantial improvement. Future work should, therefore, focus on geometric optimization of the mixer, such as modifying blade design, inclination, or spacing, and incorporating flow-guiding elements to mitigate these inherent limitations. Extending the DEM model to include the effects of moisture and liquid bridges represents another essential direction for realistically simulating spray-based conditioning processes.

In summary, this study provides a particle-scale analysis of radial mixing dynamics in a dry granular system. The findings on the critical stirring-shaft speed and the layer-dependent mixing mechanisms offer valuable guidance for the design and operation of mixing equipment in the rice processing industry and beyond, establishing a solid basis for future investigations into wet granular systems.

## 5. Conclusions

The discrete element method (DEM) model developed in this study was validated against experimental measurements of power consumption and particle volume proportions, confirming its reliability for simulating the dry granular mixing of brown rice in a U-shaped mixer. While the simulated and experimental results showed good overall agreement, minor discrepancies were attributed to the inherent stochasticity of particle-scale collisions and the simplified geometric representation of grains. This validated model was subsequently used to investigate the influence of stirring-shaft speed on radial mixing dynamics. The primary findings are as follows:

(1) A critical stirring-shaft speed of approximately 20 rpm was identified. Below this speed, radial mixing uniformity improved significantly with increasing speed. Beyond this point, further speed increases yielded negligible improvement, indicating a saturation effect in mixing effectiveness.
(2) The saturation phenomenon is explained by a dual mechanism: while higher speeds increase particle diffusion capacity, they also amplify fluctuations in particle motion, particularly in the top cascading-flow zone and the bottom layer. These intensified instabilities counteract the benefits of enhanced diffusion.
(3) At a constant speed, mixing homogeneity was consistently lower in the top and bottom layers compared to intermediate layers, highlighting inherent geometric limitations that cannot be overcome by speed adjustment alone.
(4) The study clarifies that while the DEM analysis focused on dry mixing, the insights into radial transport mechanisms provide a critical theoretical foundation for optimizing related industrial processes (e.g., spray conditioning) where uniform distribution is essential.

In practical terms, operating near the critical speed (20 rpm) can optimize mixing efficiency while minimizing energy consumption. Future work should focus on geometric modifications of the mixer to address the persistent non-uniformity in top and bottom regions.

## Figures and Tables

**Table 1:** Parameters used in simulation.

Parameter	Value
Poisson’s ratio of brown rice particle	0.4
Particle density of brown rice/kg·m^−3^	1086
Shear modulus of brown rice particle/Pa	1.1 × 10^7^
Coefficient of restitution (particle–particle) for brown rice	0.6
Coefficient of restitution (particle–wall) for brown rice	0.6
Static friction coefficient (particle–particle) for brown rice	0.43
Static friction coefficient (particle–wall) for brown rice	0.3
Dynamic friction coefficient (particle–particle) for brown rice	0.01
Dynamic friction coefficient (particle–wall) for brown rice	0.01
Density of U-shaped container/kg·m^−3^	7800
Poisson’s ratio of U-shaped container	0.3
Shear modulus of U-shaped container/Pa	7 × 10^10^

## Data Availability

The original contributions presented in this study are included in the article. Further inquiries can be directed to the corresponding author.
